# Prediction of Readmission and Complications After Pituitary Adenoma Resection via the National Surgical Quality Improvement Program (NSQIP) Database

**DOI:** 10.7759/cureus.14809

**Published:** 2021-05-02

**Authors:** Joshua Hunsaker, Majid Khan, Serge Makarenko, James Evans, William Couldwell, Michael Karsy

**Affiliations:** 1 Neurological Surgery, University of Utah, Salt Lake City, USA; 2 Medicine, Reno School of Medicine, University of Nevada, Reno, USA; 3 Neurosurgery, Thomas Jefferson Medical College, Philadelphia, USA

**Keywords:** pituitary, adenoma, complication, readmission, national surgical quality improvement program, nsqip

## Abstract

Introduction

Pituitary adenomas are common intracranial tumors (incidence 4:100,000 people) with good surgical outcomes; however, a subset of patients show higher rates of perioperative morbidity. Our goal was to identify risk factors for postoperative complications or readmission after pituitary adenoma resection.

Methods

We undertook a retrospective cohort study of patients who underwent surgery for pituitary adenoma in 2006-2018 by using the National Surgical Quality Improvement Program database. The main outcome measures were patient complications and the 30-day readmission rate.

Results

Among the 2,292 patients (mean age 53.3±15.9 years), there were 491 complications in 188 patients (8.2%). Complications and 30-day readmission have remained stable over time rather than declined. Unplanned readmission was seen in 141 patients (6.2%). Multivariable analysis demonstrated that hypertension (OR=1.6; 95% CI= 1.1, 2.1; p=0.005) and high white blood cell count (OR=1.08; 95% CI=1.03, 1.1; p=0.0001) were independent predictors of complications. Return to the operating room (OR=5.9, 95% CI=1.7, 20.2, p=0.0005); complications (OR=4.1, 95% CI=1.6, 10.6, p=0.004); and blood urea nitrogen (OR=1.08, 95% CI=1.02, 1.2, p=0.02) were independent predictors of 30-day readmission.

Conclusion

Using one of the largest datasets of pituitary adenoma patients, we identified perioperative factors most critical for patient outcome. One strength of this study is adjusting for cofactors that predict outcomes, which has not been done previously. Several patient biomarkers, namely white blood cell count and blood urea nitrogen, may serve as preoperative markers that might identify patients at higher risk. Control of blood pressure and renal disease may be perioperative management strategies that can impact the outcome.

## Introduction

Tumors of the pituitary gland are the second most common intracranial tumor (incidence 4:100,000 people); their apparent incidence has increased over the past 30 years, likely because of advances in and accessibility of intracranial imaging as well as aging of the population [1-3]. Recent surgical trends have favored the endoscopic removal of pituitary tumors as an alternative to microscopic resection [4-6]. The prediction of peri- and postoperative outcomes has been emphasized in recent years with the increasing availability of electronic medical data, more formalized training for resection, objective measures of clinical outcomes, and improved understanding of the complication rates in pituitary adenoma resections [7-9]. Surgical databases offer the ability to review national data over long periods of time and identify subgroups of patients achieving better patient outcomes.

The purpose of this study was to identify the risk factors involved in the development of postoperative complications or readmission within 30 days after undergoing resection of a pituitary adenoma by using a national surgical database.

## Materials and methods

Data source

The American College of Surgeons National Surgical Quality Improvement Program (NSQIP) registry data from 2006 through 2018 were used for this study. The dataset provides perioperative data collected and recorded for the first 30 days after surgery at 400 hospitals that participate in the program throughout the U.S. [10]. The data are collected by trained research coordinators following an established protocol, and the database undergoes periodic data quality control. The common procedural terminology (CPT) codes 61546 (craniotomy for hypophysectomy or excision of pituitary tumor, intracranial approach), 61548 (hypophysectomy or excision of pituitary tumor, transnasal or transseptal approach, nonstereotactic), and 62165 (neuroendoscopy, intracranial; with excision of pituitary tumor, transnasal or trans-sphenoidal approach) were used for this study, and all patients with available variables were included. These review codes are the most consistent with identifying pituitary adenomas but are limited as they do not account for unlisted codes or other variations in practice that may be used to code for pituitary adenomas nationally. Institutional review board approval is not necessary because the data are de-identified upon entering into the registry.

Variables and outcomes

Missing variables were excluded during the tabulation of results. The primary outcomes were perioperative complications that occurred during the patient’s admission and 30-day readmission rates, with each variable being distinctly coded in the database. A variable for “any complication” was generated using the prescribed variables in NSQIP except for the return to the operating room or 30-day readmission. Complications and return to the operating room were identified during the index surgery while 30-day readmission was after discharge and is a discrete coded variable in the NSQIP database. Patient demographic, biomarker, and clinical data were collected for classification.

Statistical analysis

Data compilation was performed with Orange software (University of Ljubljana; https://orange.biolab.si/), and statistical analysis was performed with SPSS V24.0 (IBM, Armonk, NY). T-test and Chi-square test were used for continuous and discrete variables, respectively. Linear and logistic multivariate regression was performed, with variables from the univariate analysis showing a p<0.2 entered into the forward-model likelihood ratio multivariate model. A p-value <0.05 was considered significant, but in light of the higher likelihood of type I errors with statistical tests in large databases, evaluation of effect size and confidence intervals was performed where appropriate. The Strengthening the Reporting of Observational Studies in Epidemiology (STROBE) guidelines were used in the drafting of this paper.

## Results

Demographics

**Figure 1:**
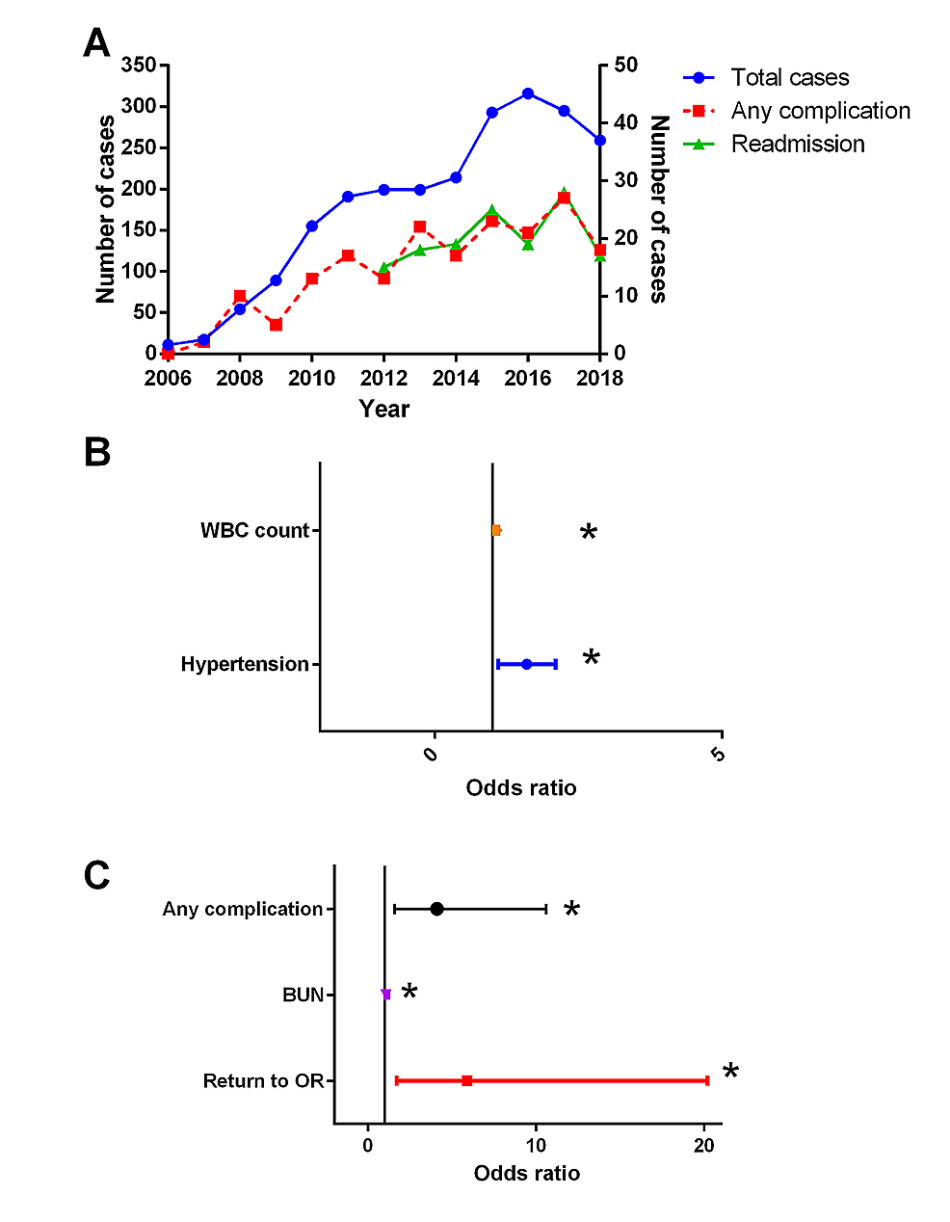
Evaluation of patients who underwent pituitary adenoma surgery. (A) Total cases, cases with any complication, and cases with readmission from 2006 through 2018 remained stable over time. (B) Multivariate logistic regression analysis of perioperative complication rate identified that preoperative WBC count, return to the operating room, and hypertension were significant independent predictors. (C) Multivariate logistic regression analysis of 30-day readmission demonstrated that any perioperative complication, BUN level, and return to the operating room were independently associated with the outcome. The odds ratio and 95% confidence interval are represented. WBC - white blood cell BUN - blood urea nitrogen

A total of 2,292 patients (50% male) treated from 2006 through 2018 were identified from the NSQIP database (Table 1, Figure 1A). Most cases were identified by CPT code 61548 (86.7%); the rest were coded by 61546 (13.3%), and no cases were coded 62165. The average age of patients was 53.3±15.9 years. Hypertension (45.2%) and diabetes (17.0%) were common comorbidities. The majority of patients were admitted from (88.2%) and discharged to (80.8%) home. The mean length of stay was 4.9±6.6 days (median=3.0 days).

**Table 1:** Demographics, comorbidities, and perioperative characteristics of 2,292 patients who underwent pituitary adenoma surgery.

Variable	Number (%) or mean ± SD
Age, years	53.3±15.9
Sex, male	1,146 (50 %)
Race	
American Indian	7 (0.3%)
Asian	161 (7.0%)
Black	330 (14.4%)
Native Hawaiian/Pacific Islander	7 (0.3%)
White	1,413 (61.6%)
Ethnicity, Hispanic	180 (7.9%)
Body mass index, mg/kg^2^	31.0±8.1
Comorbidities	
Diabetes	389 (17.0%)
Smoker	316 (13.8%)
Dyspnea	101 (4.4%)
Chronic obstructive pulmonary disease	44 (1.9%)
Hypertension	1,036 (45.2%)
Cancer	36 (1.6%)
Open wound/wound infection	10 (0.4%)
Chronic steroid use	246 (10.7%)
Unexpected weight loss	32 (1.4%)
Bleeding disorder	11 (0.5%)
Preoperative systemic sepsis	26 (1.1%)
Emergency surgery	58 (2.5%)
Operative time, minutes	160.5±95.4
Return to operating room	91 (4.0%)
Length of stay, days	4.9±6.6
American Society of Anesthesiologists class	
No disturbances	58 (2.5%)
Mild	935 (40.8%)
Severe	1,178 (51.4%)
Life-threatening	113 (4.9%)
Admission source	
Acute hospital	47 (2.1%)
Admitted from home	2,021 (88.2%)
Nursing home	8 (0.3%)
Outside emergency room	36 (1.6%)
Other	7 (0.3%)
Unknown	173 (7.5%)
Discharge destination	
Home	1,852 (80.8%)
Rehabilitation	47 (2.1%)
Skilled nursing facility	32 (1.4%)
Unknown	361 (15.8%)

Univariate analysis of complications 

Of the 2,292 patients included in the study, 188 (8.2%) experienced at least one complication within 30 days of the principal operative procedure (Table 2, Figure 1A) and a total of 141 (6.2%) had 30-day readmission (Table 3). The relative ratio of complications and 30-day readmission to overall case volume has remained stable over time. Patients were more likely to have a complication if they were older (58.3±15.4 years vs. 52.9±15.9, p=0.002) or had diabetes (26.6% vs. 16.1%, p=0.002), hypertension (56.4% vs. 44.2%, p=0.001), an open wound/wound infection at the time of the principal operative procedure (2.1% vs. 0.3%, p=0.0001), a bleeding disorder (1.6% vs. 0.4%, p=0.04), or preoperative systemic sepsis (3.7% vs. 0.9%, p=0.005). Preoperative chronic steroid use was also associated with complications (15.4% vs. 10.3%, p=0.03). Other factors associated with complications included emergency surgery (23.4% vs. 11.6%, p=0.0001), return to the operating room (21.8% vs. 2.4%, p=0.0001), higher American Society of Anesthesiologist (ASA) class (p=0.0001), and non-home admission source (p=0.0001). Of note, preoperative laboratory values that were associated with a higher risk of complications included lower serum albumin (3.9±0.5 g/dL vs. 4.1±0.5, p=0.0001) and hematocrit (39±5 vs. 40±4.3 %, p=0.0001), as well as higher alkaline phosphatase (85±41 vs. 78±29 IU/L, p=0.04), and white blood cell (WBC) count (9±5 vs. 8±3 ×109 cell/L, p=0.002).

**Table 2:** Evaluation of complications and 30-day readmission in patients who underwent pituitary adenoma surgery. *Effect size: continuous variables (mean difference with 95% confidence interval); discrete variables (Cramer’s V)

Variable	Complications				Readmission			
	None (n=2104)	Any (n=188)	Effect size*	P-value	No (n=1628)	Yes (n=141)	Effect size*	P-value
Age, years	52.9±15.9	58.3±15.4	-5.4 (‑8.8, ‑2.0)	0.002	53.1±15.9	56.2±15.4	-3.2 (-6.6, 0.3)	0.07
Year							0.06	0.7
Sex, male	1056 (50.2%)	90 (47.9%)	0.01	0.8	812 (49.9%)	75 (53.2%)	0.02	0.5
Race			0.06	0.3			0.05	0.04
American Indian	6 (0.3%)	1 (0.5%)			2 (0.1%)	2 (1.4%)	0.05	0.04
Asian	151 (7.2%)	10 (5.3%)			123 (7.6%)	9 (6.4%)		
Black	292 (13.9%)	38 (20.2%)			232 (14.3%)	18 (12.8%)		
Native Hawaiian/ Pacific Islander	7 (0.3%)	0 (0.0%)			4 (0.2%)	1 (0.7%)		
White	1305 (62.0%)	108 (57.4%)			1023 (62.8%)	85 (60.3%)		
Ethnicity, Hispanic	165 (7.8%)	15 (8.0%)	0.03	0.6	161 (9.9%)	17 (21.1%)	0.05	0.4
Body mass index, mg/kg^2^	30.9±7.9	31.8±9.6	-0.9 (-2.1, 0.3)	0.1	31.1±8.3	31.7±7.3	-0.6 (-2.0, 0.9)	0.4
Comorbidities								
Diabetes	339 (16.1%)	50 (26.6%)	0.08	0.002	295 (18.1%)	32 (22.7%)	0.03	0.2
Smoker	285 (13.5%)	31 (16.5%)	0.02	0.3	215 (13.2%)	14 (9.9%)	0.03	0.3
Dyspnea	92 (4.4%)	9 (4.8%)	0.006	0.7	57 (3.5%)	7 (5.0%)	0.02	0.4
Chronic obstructive pulmonary disease	39 (1.9%)	5 (2.7%)	0.02	0.4	31 (1.9%)	2 (1.4%)	0.01	0.7
Hypertension	930 (44.2%)	106 (56.4%)	0.07	0.001	727 (44.7%)	71 (50.4%)	0.03	0.2
Cancer	30 (1.4%)	6 (3.2%)	0.04	0.06	29 (1.8%)	4 (2.8%)	0.02	0.4
Open wound/wound infection at time of principal procedure	6 (0.3%)	4 (2.1%)	0.08	0.0001	4 (0.2%)	1 (0.7%)	0.02	0.3
Chronic steroid use	217 (10.3%)	29 (15.4%)	0.05	0.03	176 (10.3%)	18 (12.8%)	0.02	0.4
Unexpected weight loss	27 (1.3%)	5 (2.7%)	0.03	0.1	22 (1.4%)	0 (0.0%)	0.03	0.2
Bleeding disorder	8 (0.4%)	3 (1.6%)	0.07	0.04	9 (0.6%)	2 (1.4%)	0.04	0.4
Preoperative systemic sepsis	19 (0.9%)	7 (3.7%)	0.07	0.005	18 (1.1%)	1 (0.7%)	0.01	0.9
Emergency surgery	244 (11.6%)	44 (23.4%)	0.1	0.0001	239 (14.7%)	21 (14.9%)	0.002	0.9
Operative time, minutes	154.6±86.6	226.8±149.7	-72.2 (‑86.1, ‑58.2)	0.0001	161.6±97.4	183.5±116.9	-21.9 (‑41.9, ‑1.9)	0.03
Return to operating room					41 (2.5%)	29 (20.6%)	0.3	0.0001
Length of stay, days					4.4±9.5	5.0±4.1	-0.2 (-1.3, 0.9)	0.4
American Society of Anesthesiologists class				0.0001			0.06	0.001
No disturbances	58 (2.8%)	0 (0.0%)			42 (2.6%)	1 (0.7%)		
Mild	884 (42.0%)	51 (27.1%)			652 (40.0%)	58 (41.1%)		
Severe	1068 (50.8%)	110 (58.5%)			856 (52.6%)	68 (48.2%)		
Life-threatening	89 (4.2%)	24 (12.8%)			76 (4.7%)	12 (8.5%)		
Admission source			0.1	0.0001			0.05	0.5
Acute hospital	37 (1.8%)	10 (5.3%)			31 (1.9%)	6 (4.3%)		
Admitted from home	1871 (88.9%)	150 (79.8%)			1552 (95.3%)	132 (93.6%)		
Nursing home	6 (0.3%)	2 (1.1%)			7 (0.4%)	1 (0.7%)		
Outside emergency room	28 (1.3%)	8 (4.3%)			29 (1.8%)	2 (1.4%)		
Other	7 (0.3%)	0 (0.0%)			7 (0.4%)	0 (0.0%)		
Unknown	155 (7.4%)	18 (9.6%)			2 (0.1%)	0 (0.0%)		
Discharge destination							0.06	0.1
Home					1539 (94.5%)	132 (93.6%)		
Rehab					34 (2.1%)	6 (4.3%)		
Skilled nursing facility					24 (1.5%)	3 (2.1%)		
Unknown					31 (1.9%)	0 (0.0%)		
Complication					100 (6.1%)	40 (28.4%)	0.2	0.0001
Preoperative lab values								
Sodium, mEq/L	139±3	140±4	-0.1 (-0.6, 0.4)	0.6	140±3	139±4	0.5 (-0.2, 1.1)	0.3
Blood urea nitrogen, md/dL	15±8	16±7	-0.9 (-2.2, 0.5)	0.2	15±8	17±7	-1.7 (‑3.0, 0.3)	0.7
Creatinine, mg/dL	0.9±0.3	0.9±0.3	-0.01 (‑0.06, 0.04)	0.6	0.9±0.3	0.9±0.3	-0.01 (-0.07, 0.05)	0.6
Albumin, g/dL	4.1±0.5	3.9±0.5	0.2 (0.1, 0.4)	0.0001	4.1±0.5	4.0±0.4	0.04 (-0.08, 0.2)	0.04
Bilirubin, mg/dL	0.6±0.7	0.7±1.4	-0.1 (-0.4, 0.2)	0.4	0.6±0.5	0.6±0.3	0.02 (-0.1, 0.1)	0.8
Serum glutamic-oxaloacetic transaminase, U/L	26±16	30±31	3.2 (-10, 2.6)	0.2	27±18	28±36	-1.6 (-6.8, 3.6)	0.6
Alkaline phosphatase, IU/L	78±29	85±41	-7 (-13, ‑0.5)	0.04	80±32	72±24	7.2 (-1.1, 15.6)	0.09
White blood cell, 10^9^ cell/L	8±3	9±5	-1.0 (-1.7, ‑0.4)	0.002	7.8±3.3	8.4±3.4	-0.7 (-1.3, ‑0.1)	0.02
Hematocrit, %	40±4.3	39±5	1.5 (0.7, 2.3)	0.0001	40±5	40±4	-0.2 (-1.0, 0.6)	0.6
Platelet, 10^9^ cell/L	249±65	250±83	-1.3 (-11.5, 9.0)	0.8	249±67	251±66	6.0 (-14.4, 9.0)	0.7
Partial thromboplastin time, seconds	30±5	30±5	0.3 (-0.5, 1.2)	0.4	30±5	29±5	0.6 (-0.04, 0.05)	0.8
International normalized ratio	1±0.2	1±0.1	-0.01 (‑0.05, 0.02)	0.5	1±0.3	1±0.1	0.006 (‑0.04, 0.05)	0.8
Prothrombin time, seconds	12±2	12±1	0.2 (0.3, ‑0.5)	0.6	13±2	13±2	-0.2 (-2.7, 2.2)	0.8

**Table 3:** Thirty-day readmission causes

ICD10 code	ICD10 description	Number of patients (%)
	Unknown	54 (2.36)
	Other (list ICD 10 code)	50 (2.18)
E22.2	Syndrome of inappropriate secretion of antidiuretic hormone	7 (0.31)
G96.0	Cerebrospinal fluid leak	7 (0.31)
A41.9	Sepsis	5 (0.22)
E87.1	Hypo-osmolality and hyponatremia	4 (0.17)
G93.89	Other specified disorders of brain	2 (0.09)
R04.0	Epistaxis	2 (0.09)
T81.89XA	Other complications of procedures	2 (0.09)
A04.72	Clostridium difficile colitis	1 (0.04)
C79.51	Secondary malignant neoplasm of bone	1 (0.04)
E11.65	Type 2 diabetes mellitus with hyperglycemia	1 (0.04)
E24.0	Pituitary-dependent Cushing's disease	1 (0.04)
E27.9	Disorder of adrenal gland, unspecified	1 (0.04)
G44.89	Other headache syndrome	1 (0.04)
E23.2	Diabetes insipidus	1 (0.04)
Z51.11	Encounter for antineoplastic chemotherapy	1 (0.04)
R55	Syncope and collapse	1 (0.04)

In terms of complication profile, a total of 491 complications were identified in 188 patients. Complication rates ranged from 0.3% to 2%, with need for blood transfusion (1.8%), urinary tract infection (1.5%), and unplanned reintubation (1.5%) being the most common. Other complications included failure to wean from ventilation (1.2%), stroke (1.2%), pneumonia (1.0%), sepsis (1.0%), pulmonary embolism (0.7%), cardiac arrest (0.3%), and septic shock (0.3%). The overall rate of mortality was 0.8%. Rates of major complications, however, remain relatively low.

Univariate analysis of 30-day readmission 

A total of 141 patients (6.2%) underwent unplanned readmission within 30 days postoperatively, with 116 readmissions related to the original surgery (Table 2). Readmission was associated with race (p=0.04), as well as higher operative time (183.5±116.9 vs. 161.6±97.4 minutes, p=0.03), return to operating room after the principal surgery (20.6% vs. 2.5%, p=0.0001), higher ASA class (p=0.001), and the presence of complications (28.4% vs. 6.1%, p=0.0001). The only laboratory abnormality associated with higher readmission was a higher preoperative WBC count (8.4±3.4 vs. 7.8±3.3×109 cell/L, p=0.02).

Multivariate models of complication and 30-day readmission

Complication (Table 4, Figure 1B) and 30-day readmission (Table 4, Figure 1C) rates were evaluated after adjusting for other covariates. On multivariate analysis, factors associated with complications were hypertension (odds ratio (OR)=1.6; 95% confidence interval (CI)=1.1, 2.1; p=0.005) and WBC count (OR=1.08; 95% CI=1.03, 1.1; p=0.0001) after adjusting for other relevant variables. Evaluation of 30-day readmission showed increased risk was associated with return to the operating room during principal surgery (OR=5.9; 95% CI=1.7, 20.2; p=0.0005), complications (OR=4.1; 95% CI=1.6, 10.6; p=0.004), and blood urea nitrogen (BUN) (OR=1.08; 95% CI=1.02, 1.2; p=0.02).

**Table 4:** Multivariate analysis of complications in patients after pituitary adenoma surgery.

Variable	Complications				30-day readmission			
	Univariate analysis		Multivariate analysis		Univariate analysis		Multivariate analysis	
	P-value	OR (95% CI)	P-value	OR (95% CI)	P-value	OR (95% CI)	P-value	OR (95% CI)
Age, years	0.002	1.02 (1.01, 1.04)			0.07	1.01 (0.99, 1.03)		
Year	0.3	0.97 (0.93, 1.03)			0.6	0.98 (0.89, 1.07)		
Sex, male	0.5	0.91 (0.68, 1.23)			0.5	1.14 (0.81, 1.6)		
Race								
American Indian	0.5	2.01 (0.24,16.89)			0.01	12.04 (1.67, 86.51)		
Asian	0.5	0.80 (0.41, 1.57)			0.7	0.88 (0.43, 1.8)		
Black	0.02	1.57 (1.06, 2.32)			0.8	0.93 (0.55, 1.58)		
Native Hawaiian/ Pacific Islander	0.99	-			0.3	3.01 (0.33, 27.22)		
White	0.3	Reference			0.1	Reference		
Ethnicity, Hispanic	0.8	1.07 (0.6, 1.92)			0.2	1.42 (0.81, 2.51)		
Body mass index, mg/kg^2^	0.1	1.01 (0.99, 1.03)			0.4	1.008 (0.99, 1.03)		
Comorbidities								
Diabetes	0.0001	1.90 (1.35, 2.68)			0.2	1.33 (0.88, 2.01)		
Smoker	0.3	1.26 (0.84, 1.89)			0.3	0.72 (0.41, 1.28)		
Dyspnea	0.8	1.1 (0.55, 2.22)			0.4	1.44 (0.64, 3.22)		
Chronic obstructive pulmonary disease	0.4	1.46 (0.56, 3.72)			0.7	0.74 (0.18, 3.13)		
Hypertension	0.001	1.63 (1.23, 2.21)	0.005	1.6 (1.1, 2.1)	0.2	1.26 (0.89, 1.77)		
Cancer	0.07	2.29 (0.94, 5.55)			0.4	1.60 (0.56, 4.65)		
Open wound/wound infection	0.002	7.60 (2.13, 27.18)			0.3	2.90 (0.32, 26.12)		
Chronic steroid use	0.03	1.50 (1.04, 2.41)			0.4	1.28 (0.76, 2.15)		
Unexpected weight loss	0.1	2.10 (0.8, 5.52)			.99	-		
Bleeding disorder	0.03	4.66 (1.21, 17.84)			0.2	2.71 (0.58, 12.72)		
Systemic sepsis	0.001	4.23 (1.76, 10.21)			0.7	0.64 (0.09, 4.82)		
Emergency surgery	0.04	2.12 (1.02, 4.36)			0.9	0.96 (0.29, 3.16)		
Operative time, minutes	0.0001	1.005 (1.004, 1.007)			0.01	1.002 (1.000, 1.003)		
Return to the operating room					0.0001	10.02 (6.00, 16.74)	0.005	5.9 (1.7, 20.2)
Length of stay, days					0.7	1.005 (0.98, 1.03)		
American Society of Anesthesiologists class								
No disturbances	0.99	-			0.07	0.15 (0.02, 1.20)		
Mild	0.0001	0.21 (0.13, .36)			0.09	0.56 (0.29, 1.1)		
Severe	0.0001	0.38 (0.23, .62)			0.04	0.50 (0.26, 0.97)		
Life-threatening	0.0001	Reference			0.1	Reference		
Admission source								
Acute hospital	0.05	2.33 (0.99, 5.46)			0.6	Reference		
Admitted from home	0.2	0.69 (0.42, 1.16)			0.07	0.44 (0.18, 1.07)		
Nursing home	0.2	2.87 (0.54, 15.3)			0.8	0.74 (0.08, 7.15)		
Outside emergency room	0.06	2.46 (0.98, 6.20)			0.2	0.33 (0.07, 1.91)		
Other	0.999	-			0.999	-		
Unknown	0.0001	Reference			0.999	-		
Discharge destination								
Home					0.4			
Rehab					0.1	2.06 (0.85, 4.99)		
Skilled nursing facility					0.5	1.46 (0.43, 4.90)		
Unknown					0.998	-		
Complication					0.0001	6.05 (3.98, 9.2)	0.004	4.1 (1.6, 10.6)
Preoperative lab values								
Sodium, mEq/L	0.6	1.01 (0.97, 1.06)			0.1	0.96 (0.9, 1.01)		
Blood urea nitrogen, md/dL	0.2	1.009 (0.995, 1.02)			0.04	1.02 (1.001, 1.04)	0.02	1.08 (1.02, 1.2)
Creatinine, mg/dL	0.6	1.1 (0.7, 1.7)			0.6	1.1 (0.7, 1.8)		
Albumin, g/dL	0.0001	0.4 (0.3, 0.6)			0.5	0.8 (0.49, 1.46)		
Bilirubin, mg/dL	0.2	1.1 (0.9, 1.4)			0.8	0.9 (0.5, 1.7)		
Serum glutamic oxaloacetic transaminase, U/L	0.07	1.008 (0.99, 1.02)			0.5	1.003 (0.9, 1.02)		
Alkaline phosphatase, IU/L	0.04	1.006 (1.000, 1.012)			0.08	0.99 (0.98, 1.001)		
White blood cell, 10^9^ cell/L	0.0001	1.07 (1.04, 1.1)	0.0001	1.08 (1.03, 1.1)	0.03	1.05 (1.006, 1.1)		
Hematocrit, %	0.0001	0.93 (0.9, 0.97)			0.6	1.009 (0.97, 1.05)		
Platelet, 10^9^ cell/L	0.8	1.000 (0.998, 1.003)			0.7	1.001 (0.998, 1.003)		
Partial thromboplastin time, seconds	0.4	0.99 (0.9, 1.02)			0.2	0.97 (0.93, 1.02)		
International normalized ratio	0.5	1.2 (0.7, 2.0)			0.8	0.88 (0.31, 2.5)		
Prothrombin time, seconds	0.6	0.95 (0.79, 1.2)			0.8	1.04 (0.7, 1.5)		

Missing data

Of the patients found to have had a return to the operating room (n=91), a total of 68 did not have the proper CPT codes or International Classification of Diseases, Ninth Revision, Clinical Modification (ICD-9-CM) codes to allow us to accurately identify the procedure performed and the specific reason. Additionally, there were 523 patients of the 2,292 that did not have a definite "readmission" or "non-readmission" code. As such, we excluded those patients from our data analysis during the analysis of risk factors impacting the readmission rate. For the multivariate analysis, similar results were obtained when performing sensitivity analysis with the removal of highly significant variables from models as well as the removal of variables with >10% missing data. Reasons for return to the operating room were often missing (n=68), but common explanations included hematoma evacuation (n=6), cerebrospinal fluid (CSF) leak (n=4), resection of additional tumor (n=8) and other unrelated procedures (n=5). Similarly, reasons for 30-day readmission were commonly absent (n=50) or unknown (n=54).

## Discussion

Study findings

The results of this study suggest that multiple factors have a significant influence on complication rates or 30-day readmission after the resection of pituitary adenomas. Among all of the evaluated factors, preoperative hypertension and elevated WBC count were significant predictors of perioperative complications. These are notable as they may be potentially modifiable risk factors. Furthermore, preoperative BUN levels, perioperative complications, and return to the operating room were significant predictors of 30-day readmission. Interestingly, operative time and ASA classification were important factors for complications and 30-day readmission but were not important in the multivariate analysis. Surprisingly, complications and 30-day readmission rates have remained stable over time rather than declined despite improved surgical techniques and standardized treatment strategies.

Clinical factors

These results suggest that some modifiable factors are associated with patient outcomes (e.g., hypertension, WBC, BUN), but it is unclear how better management can improve patient outcomes. Preoperative serological biomarkers, namely WBC count and BUN levels, were the most promising for predicting outcome and could be objective metrics of underlying patient health. WBC count, and specifically neutrophil-to-lymphocyte ratio, has been shown to be an important factor in predicting outcomes for a variety of diseases, including head trauma [11], subarachnoid hemorrhage [12,13], and tumors [14,15]. Clinically, high levels of BUN preoperatively could be an indication of kidney dysfunction and be a major contributing factor to the development of postoperative urinary tract infections, which was one of the most common complications in our cohort. High levels of BUN have also been shown to be a risk factor for the development of pneumonia after surgery [16]. Similarly, return to the operating room played an important role in 30-day readmission (OR=4.1), but it is unclear how preventable this is. While some return to the operating room may be unavoidable, the clear association of repeat early surgery and poor outcomes should be notable because it highlights the importance of the surgeon and treatment team in patient care. The lack of improvement in complications and 30-day readmission also suggests additional room for patient outcome improvement.

Patient complication after pituitary adenoma surgery

Multiple single-center database studies and meta-analysis reviews have evaluated outcomes after pituitary adenoma surgery. Agam et al. [6] evaluated 1,153 consecutive patients undergoing trans-sphenoidal microscopic and endoscopic approaches for pituitary adenoma resection. The authors noted a median hospital stay of three days, perioperative death rate of 0.1%, an overall complication rate of 17.0%, and surgical complication rate of 6.8%. The most common surgical and medical complications were postoperative CSF leak (2.6%) and bacteremia/sepsis (0.5%), respectively. Microscopic and endoscopic approaches showed no differences in surgical complications (6.4% vs. 8.8%, p=0.2) or endocrinological complications (11.4% vs. 11.8%, p=0.9). Risk factors for complications were prior transsphenoidal surgery, preoperative vision loss, and the presence of invasion on MRI. These results reflect the low overall complication rate of patients undergoing pituitary tumor surgery and delineate between different surgical approaches. The 30-day readmission rate was not addressed, but high-risk features on imaging during pituitary adenoma resection were notable. Limitations of these data include the retrospective nature of the data analysis and limited external validity due to the study predominantly including data from a single surgeon.

Another multicenter study evaluated 982 patients undergoing endoscopic pituitary surgery at six international centers between 2002 and 2014 [17]. This study demonstrated a median hospital stay of five days and an overall adverse event rate of 23.8%. Risk factors predicting complications included intraventricular tumor extension and previous radiation. Reoperation occurred in 6.5% of patients, with risk factors including intraventricular extension and younger age. CSF leak risk was associated with female sex, high body mass index (BMI), lower age, and intraventricular extension. These results are interesting in supporting imaging findings (i.e., intraventricular extension) that correlate with risk. The strengths of this study include its more homogeneous sample of endoscopic approaches and multiple centers; however surgical treatments were done at high-volume centers with experts in the field. Thus, the results of this study may not apply to surgeons at all centers.

Database analysis of pituitary surgery

Several studies have aimed to use available national databases to evaluate risk in pituitary surgery, namely the National Inpatient Sample [1,18-25] and NSQIP [16,21,22,26-28]. Lawrence et al. [27] evaluated 658 patients that underwent transnasal microscopic pituitary surgery between 2006 and 2012 by using the NSQIP database. An overall complication rate of 8.8% was seen, with the most common complications being reoperation (1.7%), unplanned reintubation (2.0%), urinary tract infection (1.7%), and transfusion (1.7%). Predictors of complication included preoperative sepsis and lower preoperative albumin. Younger patients were associated with a greater surgical complication rate whereas higher BMI, chronic steroid use, preoperative sepsis and lower preoperative serum hematocrit were associated with medical complications. Increased hospital length of stay was associated with older age, higher BMI, chronic steroid use, preoperative sepsis, and lower albumin. Our results were similar in identifying the perioperative factors and biomarkers that were predictive of outcome; however, our results controlled for the heterogeneity of patient data via multivariate analysis. In comparison to Lawrence et al., our data covered a larger time range (2006-2018), aimed to provide effect size for several analyzed variables, and used return to the operating room as a predictive variable for 30-day readmission rate. Most other related NSQIP studies involved anterior skull base surgery in general and thus do not specifically apply to pituitary adenomas [16,21,22,26,28].

Return to the operating room

We found that 91 patients (4.0%) had a return to the operating room after the initial surgery. CPT codes indicating the reason for reoperation were listed for 23 of the 91 patients (25.2%). Of those 23 patients, the most common reasons for reoperation were further tumor resection, CSF leak/dural repair, and hematoma. Further tumor resection was the primary reason for reoperation, which suggests a limitation of interpreting the NSQIP dataset. Although some of these patients may have been indicated for a return to the operating room to excise the residual tumor, others may have returned to the operating room for other complications or indications, with the original CPT or ICD-9 code acting as a basic proxy.

CSF leaks are among the most common complications of pituitary adenoma surgery [26]. Our data show that seven patients (30.4%) returned to the operating room because of a CSF leak or need for a dural repair as indicated by CPT codes (15570, 20937, 30520, 31287, 63707). CSF leaks can be variable, based on the surgical approach, patient factors (e.g., BMI) [22], and initial repair techniques, making them extremely difficult to predict. Strickland et al. [29] performed a meta-analysis of the literature from 1995 through 2016 evaluating CSF rhinorrhea in 1002 patients. These results showed that half of all patients (n=26) that developed a postoperative CSF leak did not have a noted intraoperative leak or empirical sellar repair.

Limitations

The main limitation of this study is the incomplete data from the NSQIP dataset that were used as the only source of patient information and outcomes for this study. Missing data points differed depending on the variable included in the database. In addition, long-term follow-up and more granular outcome data (e.g., tumor resection rate, quality-of-life measures) were not available. No cases were coded as CPT 62165 (neuroendoscopy, intracranial; with excision of pituitary tumor, transnasal or transsphenoidal approach), but numerous cases had CPT 15740 (flap; island pedicle) or 15750 (flap; neurovascular pedicle), which would only be associated with endoscopic approaches. While these approaches are fairly specific to pituitary adenoma approaches, they may not capture all the relevant patients. These data also suggest that endoscopic approaches were being performed in the NSQIP dataset but could not be distinguished from open approaches. Despite being a prospectively collected surgical database that has been broadly used in the surgical literature, further studies are needed to better account for patient heterogeneity, and potentially including imaging could aid in this problem.

NSQIP was designed to track patients for 30 days after the initial operation, which could lead to a lower number of complications than actually occurred because of a lack of long-term follow-up, with late complications being a known issue with pituitary surgery [30]. In addition, the importance of WBC, hypertension, and BUN is limited by the relatively low odds ratios for predicting outcomes. Despite available literature supporting the importance of these factors overall, more work is needed to evaluate a causal relationship with clinical factors and worsened risk with pituitary adenoma surgery. Lastly, the obvious risk factors of patient complication and return to the OR may also be challenging to modify.

Notwithstanding these limitations, this study helps to better understand the potential risk factors that can lead to a higher incidence of complications and readmission in patients undergoing surgical treatment of pituitary tumors regardless of the operative approach. Because of the nature of the NSQIP program and the dataset, there is less selection bias that can help generate more generalizable and valid results. With hundreds of hospitals around the country tracking and submitting results to this program for over a decade, this database and patient population give us the best overall picture of the comorbidities and preoperative risk factors most likely to be associated with negative outcomes in our own patients.

## Conclusions

Our results suggest that increased preoperative WBC count, return to the operating room, and hypertension were associated with increased complication rate during pituitary adenoma resections. Furthermore, complications, elevated preoperative BUN, and return to the operating room were independent predictors of 30-day readmission. WBC and BUN are potential biomarkers of risk and are readily available clinically. All of these factors can be potentially modifiable. Further work can potentially refine these and other risk factors to generate appropriate predictive models and cutoff values. Over time, complication rates and 30-day readmission after the resection of pituitary tumors have remained stable. These findings are limited by the coding of the NSQIP and missing data. The strength of this data is in the use of a prospective, curated, multicenter dataset. Understanding the effect of comorbidities on potential complications and readmission rates is vital to patient care and can present new areas of prospective investigation. The challenges for optimizing these factors in patients remain an area of ongoing research.
